# Association between Body Mass Index and Externalizing and Internalizing Symptoms among Chinese Adolescents: Mediating Role of Traditional Bullying and Cyberbullying Victimization

**DOI:** 10.3390/bs14060427

**Published:** 2024-05-22

**Authors:** Jiajun Zhou, Qingchen Da, Linlin Xie, Yifan Jiang, Liping Li

**Affiliations:** 1 School of Public Health, Shantou University, Shantou 515041, China; 22jjzhou@stu.edu.cn (J.Z.); 21qcda@stu.edu.cn (Q.D.); 22llxie@stu.edu.cn (L.X.); 20yfjiang@stu.edu.cn (Y.J.); 2 Injury Prevention Research Center, Shantou University Medical College, Shantou 515041, China

**Keywords:** obesity, overweight, body mass index, traditional bullying, cyberbullying, externalizing symptoms, internalizing symptoms

## Abstract

Background: Externalizing problems, internalizing problems, and obesity are among the greatest challenges to adolescent health. However, the moderating and mediating mechanisms that underlie this association remain predominantly unexplored. Objectives: In this study, we examined the association between body mass index (BMI) and externalizing and internalizing scores in adolescents, tested whether traditional bullying and cyberbullying mediated the association, and explored the moderated role of sex. Methods: The data came from 1486 adolescents from grade 7, 8, and 10 living in Shantou, China. Information on BMI, traditional bullying, and cyberbullying victimization was obtained through a self-administered questionnaire. The students’ externalizing and internalizing scores were evaluated using the Strengths and Difficulties Questionnaire (SDQ). Furthermore, we built two parallel mediation models with sex as a moderating variable. Results: Compared to their peers with normal weight, adolescents with increased BMI reported higher externalizing and internalizing scores. Traditional bullying and cyberbullying were both significant mediators in the two relationships. Sex moderated the pathway from BMI to cyberbullying. But sex did not moderate the relationship between BMI and traditional bullying. Conclusions: The results highlight that it is imperative for educators to identify students who are subjected to weight-based bullying and provide them with recommendations for effective coping strategies. Meanwhile, both victims of traditional bullying and those affected by cyberbullying should be the focus of prevention and intervention efforts when developing a strategy to improve levels of internalizing and externalizing symptoms among adolescents with increased BMI.

## 1. Introduction

Obesity is a significant public health issue impacting the health of Chinese adolescents. China has the highest number of obese adolescents globally [1]. Over recent decades, obesity rates have markedly increased. According to the Chinese National Survey on Students’ Constitution-18, the overweight rate increased from 1.1% in 1985 to 12.1% in 2014, and the prevalence of obesity rose from 0.1% to 7.3% [2]. Based on the 2017 Physical Activity and Fitness in China-The Youth Study, among Chinese students aged 7–19, the rates of overweight and obesity were 15.1% and 10.7%, respectively [3]. A commonly used measure for assessing adiposity is the body mass index (BMI). In China, a person with a BMI of 24.0 to 27.9 kg/m^2^ is defined as overweight, and a BMI of 28.0 kg/m^2^ or higher is considered obese [4].

Having a high BMI not only impacts an individual’s physical health but also leads to a range of psychological and behavioral issues, categorized into two main types: internalizing and externalizing problems [5,6]. Internalizing problems refer to those that are “inner-directed and generate distress in the individual”, such as anxiety and depression [7]. Externalizing problems are “outer-directed and generate discomfort and conflict in the surrounding environment”, such as antisocial behavior and impulse control disorders [8,9]. Therefore, the connection between a high BMI and these internalizing and externalizing issues underscores the importance of BMI in an individual’s psychological health and behavioral development.

The association between BMI and internalizing and externalizing problems is complex and may involve indirect relationships mediated by other variables. Bullying experiences may be an important mediator in the relationship between BMI and internalizing and externalizing problems [10,11,12]. Among individuals with a higher BMI, experiences of bullying may play a crucial role in the manifestation of psychological problems [13]. Some studies have found that bullying mediates the association between BMI and specific internalizing or externalizing problems, such as depression and suicidal tendencies [14]. Moreover, C. L. van Vuuren and colleagues found that compared to their normal-weight peers, adolescents with a higher BMI reported more frequent internalizing problems such as psychosocial problems and suicidal thoughts, with bullying victimization being an important mediator in the relationship between excess weight and psychosocial issues [15]. Meanwhile, different types of bullying play different roles. Byung Lee et al. revealed that traditional bullying victimization (relational bullying, verbal bullying, property bullying, and physical bullying) mediated the association between BMI and internalized problems such as physical distress [16]. However, the mediating effect of cyberbullying was not present. Research conducted by Byung Lee et al. found that while there is a significant direct association between body mass index (BMI) and both physical and mental health, the mediating effect on internalized problems such as physical suffering is significant only through traditional bullying victimization; cyberbullying does not have a significant mediating effect [16]. In addition, the findings on whether gender moderates the relationship between BMI and internalizing or externalizing problems are mixed. A study in Taiwan on the mediating role of bullying in the relationship between BMI and social phobia, depression, suicide, and self-esteem found that gender did not exhibit a statistically significant moderating effect on the mediating role of bullying [13]. However, there is a lack of evidence from studies conducted in mainland China.

Most of the existing research has focused on the correlation between BMI and specific internalizing problems, such as depression, while a comprehensive perspective on both internalizing and externalizing problems has largely been absent. This study aims to explore the mediating role of traditional and cyberbullying victimization in the relationship between BMI and both internalizing and externalizing problems, with a particular focus on potential gender differences. The hypothesis posits that traditional and cyberbullying victimization may exert distinct mediating effects on the relationship between BMI and internalizing and externalizing problems in adolescents, with an expectation of gender differences in the mediating influence of bullying involvement.

## 2. Materials and Methods

### 2.1. Study Population and Procedure

We used data from the Chinese subset of the 2022 Global Children and Adolescent Mental Health Study (GCAMHS). The GCAMHS is a global, cross-sectional study conducted across multiple cultural and economical contexts, utilizing a self-administered questionnaire to collect information about adolescent psychosocial well-being health behavior. The data for this study were collected in June 2022 in Shantou, located in the eastern region of Guangdong Province, China. The participants were recruited from grades 7, 8, and 10, and were within the age range from 11 to 16 years old. The 9th and 12th graders were excluded because they were busy preparing for their entrance exams. Using cluster random sampling, we randomly selected two schools from the central and non-central areas of Shantou, respectively, and randomly selected all students from five classes in each of the three grades. All participants, along with their guardians, willingly consented and provided signed informed assent or consent for their involvement in this study. Subsequently, students anonymously completed these questionnaires within their classroom settings. Using standard formulas for cross-sectional studies, we aimed for a confidence level of 95%, a margin of error of 5%, and an estimated prevalence of bullying victimization of 30% (to maximize the sample size); the minimum required sample size was calculated to be approximately 323 participants. However, to account for potential non-responses and to enhance the power of our analyses, we aimed to recruit a larger sample. Ultimately, we obtained 1486 valid questionnaires, reflecting an effective response rate of 98.61%. This study was performed in line with the principles of the Declaration of Helsinki. Approval was granted by the Ethics Committee of Shantou University Medical College (SUMC-2022-040).

Inclusion criteria were as follows: (1) voluntary participation in the survey and signing of informed consent and (2) less than 18 years of age; exclusion criteria included (1) cognitive impairment or mental illness and (2) more than 10% of the questionnaire items not being answered.

### 2.2. Measurement

#### 2.2.1. Body Mass Index (BMI)

Body mass index (BMI) was calculated based on self-reported measurements of height and weight [17]. As the formula primarily aimed to calculate adult BMI, BMI percentiles were subsequently categorized into four distinct groups: (1) underweight, defined as less than the 5th percentile; (2) healthy weight, denoting those between the 5th and 85th percentile; (3) at risk of overweight—encompassing individuals between the 85th and 95th percentile; and (4) overweight, comprising those exceeding the 95th percentile. In the present analyses, the reference category used was healthy weight, encompassing individuals falling between the 5th and 85th percentiles, to investigate the impact of overweight and obesity.

#### 2.2.2. Externalizing Scores and Internalizing Scores

We focused on the measurement of two symptoms: (i) externalizing scores and (ii) internalizing scores. The children’s internalizing and externalizing scores were assessed with a self-report version of the Strength and Difficulties Questionnaire (SDQ), which is a globally recognized screening questionnaire that prompts students to report on their behaviors and emotions over the preceding six-month period [18]. The SDQ comprises 25 items categorized into 5 subscales: emotional symptoms, conduct problems, hyperactivity, peer problems, and prosocial behavior [19]. Internalizing scores (Cronbach’s α = 0.83) are computed by summing the responses to peer and emotional problems (e.g., being easily distracted and having wandering concentration; restlessness, overactivity, and an inability to stay still), whereas externalizing scores (Cronbach’s α = 0.91) are derived as the sum total of conduct problems and hyperactivity/inattention items (e.g., often loses temper; frequently engages in fights or bullying). Both scales are assessed using 5 items scored on a 3-point Likert scale (‘not true’, ‘somewhat true’, and ‘certainly true’), which are summed together to create a total score ranging from 0 to 20. Higher scores represent more difficulties. The scores for the internalizing subscale were categorized as normal (0–7), borderline (8), and abnormal (9–20). Similarly, the scores for the externalizing subscale were categorized as normal (0–8), borderline (9), and abnormal (10–20) [20,21].

#### 2.2.3. Traditional Bullying Victimization and Cyberbullying Victimization

The questionnaire was developed by the Research Center for Child Psychiatry at the University of Turku, Finland; see Supplementary File for the specific questionnaire. The participants were queried regarding their experiences of bullying within the last 6 months. The questionnaire offered the following definition for traditional bullying victimization: “Traditional bullying occurs when a student is repeatedly exposed over an extended period to negative and hurtful actions instigated by one or more fellow students”. The targeted student finds it challenging to defend themselves against such bullying behaviors. Bullying incidents might occur with varying frequency. This behavior can manifest verbally (such as name-calling and threats), physically (including hitting), or psychologically (encompassing rumors and shunning). It is bullying when someone engages in persistent, unkind, or hurtful teasing. Subsequently, we posed 11 questions to the students, inquiring about the frequency of their experiences with bullying victimization, both within and outside of the school environment, over the preceding six months. The response options included “never”, “less than once a week”, “more than once a week”, and “almost every day”, and the corresponding scores ranged from 1 to 4. The last three options were categorized as indicative of experiencing bullying to some degree. Hence, scores exceeding 11 were designated as constituting exposure to traditional bullying.

Cyberbullying victimization was defined as follows: “Cyberbullying occurs when an individual engages in a pattern of repeatedly mocking or targeting another person through online means, including email or text messages, or when someone shares unfavorable content about another person on the internet”. Similarly, we presented the students with 8 corresponding questions, including “how frequently have you experienced cyberbullying over the past six months?” to achieve the purpose of the survey. The response options were the same as those used for traditional bullying. Scores surpassing 8 were deemed indicative of experiencing cyberbullying.

### 2.3. Covariate Variables

Previous research has shown that social–demographic characteristics play a crucial role in indicating instances of bullying victimization, alongside school-related factors [22]. Thus, in this study, we used age and household economy as covariate variables. Among them, we used the question “How economically well off do you think your family is compared to other families?” to investigate the economic condition of the participants. The possible answers were as follows: (1) not well; (2) not particularly well; (3) fairly well; (4) rather well; (5) very well.

### 2.4. Statistical Analyses

Firstly, we used SPSS25.0 statistical software to compile the collected data and perform various analyses, including descriptive statistics, reliability analysis, and Pearson correlation analysis, to examine the associations among the study variables.

**Figure 1:**
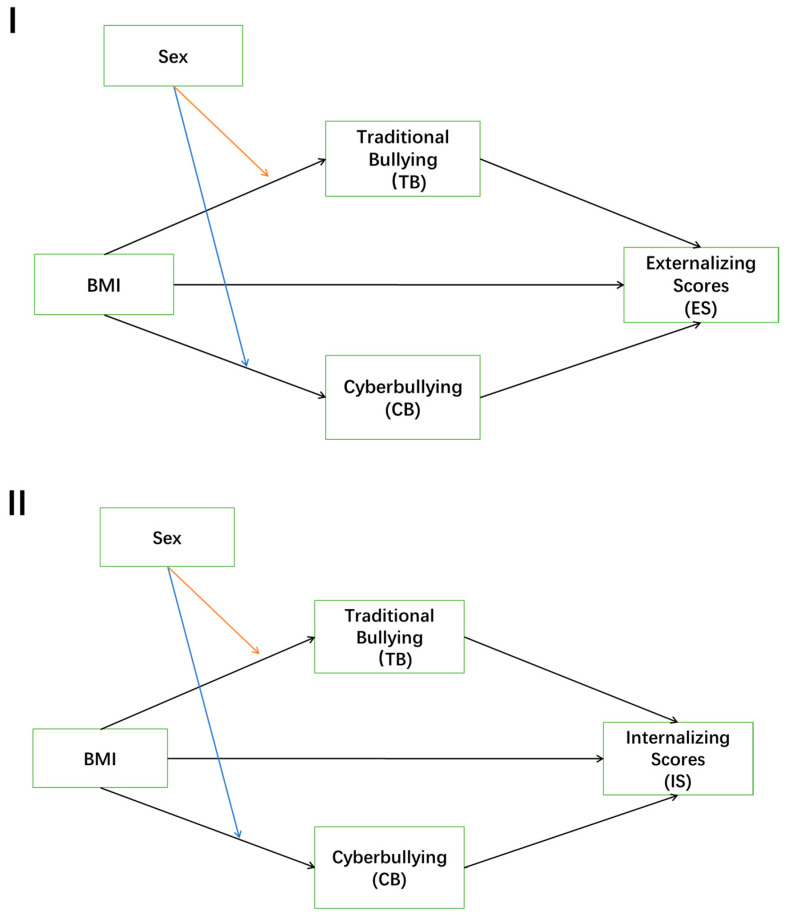
The proposed models of the associations among BMI, tradition bullying, cyberbullying, externalizing scores, and internalizing scores in adolescents (Models I and II, respectively).

Second, we analyzed the association between BMI and adolescents’ externalizing and internalizing scores, and whether these weight-based effects manifest indirectly through traditional bullying and cyberbullying. Therefore, we fit two parallel mediation models with internalizing and externalizing scores as dependent variables, respectively. The hypothesized models of the associations among them are shown in Figure 1. We then employed a bootstrapping methodology, which is recognized for its advantages over conventional approaches in testing mediation, to compute bias-corrected bootstrap confidence intervals (CIs) through bootstrap resampling. A significant indirect effect was confirmed when the 95% bias-corrected CIs, based on 5000 bootstrap samples, did not include 0. The indirect effects were calculated using the standardized regression coefficients of the study variables [23].

Finally, another objective of this study was to examine the moderating effect of sex on the mediating role of traditional bullying and cyberbullying in the relationship between BMI and externalizing scores and internalizing scores. There are two parallel mediation models as shown in Figure 1, and we introduced the moderator variable into the model and tested for a moderating effect of sex on the direct effect of BMI on traditional bullying and cyberbullying, as well as on the indirect effect of BMI on externalizing scores and internalizing scores via traditional bullying and cyberbullying. All continuous variables were standardized before the analysis. The age of the children and their household economy were controlled in all analyses.

The two models were performed in Mplus version 8.3, using full-information maximum likelihood to address missing data.

## 3. Results

### 3.1. Description of Participants

A total of 1486 students from 3 grades were included in our study. We reported summary statistics for externalizing and internalizing scores, traditional bullying victimization and cyberbullying victimization, moderator variables, and other control variables used in this study (Table 1). The participants were evenly distributed between boys and girls (boys, 53.16%; girls, 46.84%) and across grade levels (7th, 31.43%; 8th, 34.19%; 10th, 34.38%).

### 3.2. Prevalence of Traditional Bullying Victimization and Cyberbullying Victimization

In this survey, 284 out of 1486 students (19.11%) reported experiencing traditional bullying, while 387 students (26.04%) reported being victims of cyberbullying. The mean for the total traditional bullying victimization scores of the participants was 14.15, and the mean total score was 13.08 for cyberbullying victimization.

### 3.3. Bivariate Correlations

The normality test for all of the study variables found that all of the study variables obeyed normal distribution. And the correlations for all variables are presented in Table 2. As anticipated, there was a significant correlation between BMI and both externalizing (r = 0.592) and internalizing scores (r = 0.556). Additionally, traditional bullying victimization and cyberbullying victimization were positively correlated with externalizing scores (r = 0.476 and 0.603, respectively) and internalizing scores (r = 0.454 and 0.554, respectively). Moreover, BMI was positively associated with traditional bullying victimization and cyberbullying victimization (r = 0.608 and 0.732, respectively).

### 3.4. The Mediating Effects of Traditional Bullying Victimization and Cyberbullying Victimization

Table 3 show the results for the mediation effect of the two models.

In the two models, the effect of BMI on traditional bullying victimization and cyberbullying victimization was significant (β = 0.612 and *p* < 0.001; β = 0.470 and *p* < 0.001, respectively). In model I, the results indicated that after controlling for age and household economy, BMI had a significant positive effect on the prediction of internalizing scores (β = 0.399 and *p* < 0.001). The effects of both types of bullying victimization on internalizing scores were significant (β = 0.175 and *p* < 0.001; β = 0.111 and *p* < 0.001, respectively). The bootstrap results with 5000 resamples showed standardized indirect effects of 0.107 and 0.052, respectively, and both 95% confidence intervals excluded 0 (95% CI [0.076, 0.142] and [0.037, 0.067], respectively). Therefore, the results suggest that both types of bullying victimization mediated the effect of BMI on internalizing scores.

Similarly, in model II, after controlling for the effect of the two variables, BMI exhibited a significant positive association with externalizing scores (β = 0.409 and *p* < 0.001). Simultaneously, both of the two types of bullying victimization were positively and significantly associated with externalizing scores (β = 0.157 and *p* < 0.001; β = 0.194 and *p* < 0.001, respectively). And the two indirect effects were significant (95% CI [0.059, 0.137] and [0.067, 0.117], respectively). Thus, in model II, both types of bullying victimization mediated the effect of BMI on externalizing scores. See Table 3 for specific individual path coefficients.

The results of both models showed that adolescents with a higher BMI were more likely to experience traditional bullying victimization and cyberbullying victimization, which led to more externalizing and internalizing problems.

### 3.5. Moderated Mediation Analyses

After controlling students’ age and household economy, we then tested the moderating effect of sex. The results are shown in Table 4, indicating that in both models, the interaction term between BMI and sex had a significant negative predictive association with cyberbullying victimization (β = −0.117 and *p* = 0.013), signifying that sex moderated the relationship between BMI and cyberbullying victimization. However, the predictive effect of the interaction term between BMI and sex on traditional bullying victimization was not significant (β = −0.046 and *p* < 0.05), suggesting that sex did not moderate the relationship between BMI and traditional bullying victimization. Table 5 shows the moderated mediation effects of these models.

**Figure 2:**
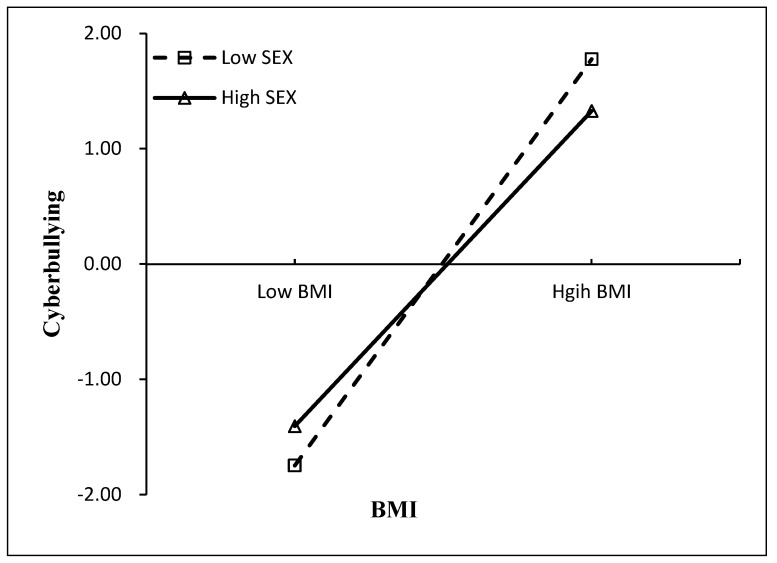
The interaction between BMI and sex on adolescents’ cyberbullying victimization.

As is presented in Figure 2, subsequent analyses of simple slopes of BMI at lower (1 SD below the mean) and higher (1 SD above the mean) levels of sex were performed to elucidate the interaction nature. In both models, among students with a low level (−1 SD) of sex, i.e., girls, BMI was positively associated with increased cyberbullying victimization. Conversely, among students with a high level of sex (+1 SD), i.e., boys, the relationship between BMI and cyberbullying victimization was relatively weaker. Therefore, being a boy appeared to function as a protective factor, while being a girl was indicative of a risk factor in the relationship between BMI and cyberbullying victimization.

## 4. Discussion

Obesity has been identified as a major risk factor for externalizing and internalizing problems in adolescence. Understanding the underlying mechanisms that could buffer the negative effects of externalizing and internalizing problems is crucial for protecting adolescents’ mental health. In this study, we aimed to investigate the associations between BMI and externalizing and internalizing scores, and to test the mediation effects of traditional bullying and cyberbullying as well as the moderation effects of sex.

### 4.1. BMI and Externalizing Scores and Internalizing Scores

Our study showed that BMI is significantly associated with externalizing and internalizing scores, consistent with previous research [24,25,26,27]. For example, in a study with data from 1254 children in the National Institute of Child Health and Human Development Study, a higher BMI was correlated with an elevated likelihood of developing internalizing problems [26]. Similarly, in a large representative sample of Dutch youths, BMI was associated with both internalizing and externalizing problems [25].

### 4.2. Traditional Bullying Victimization and Cyberbullying Victimization as Mediators

Mediation analysis found that both traditional bullying victimization and cyberbullying victimization played mediating roles. Thus, this finding suggests that traditional bullying victimization and cyberbullying victimization may serve as important explanatory mechanisms for the link between BMI and externalizing and internalizing scores. The mediating effects are consistent with previous research [16,28]. One plausible explanation is that the more adolescents deviate from the norm in terms of body size, the more they are bullied, and the stronger the association with externalizing and internalizing symptoms [29]. In concrete terms, it is plausible that obesity is perceived not merely as a fortuitous physical trait, but rather as an attribute imbued with implicit social components that potentially foster stigmatization, leading to greater dislike and exclusion by peers and consequent bullying [30,31,32]. Simultaneously, the theory of learned helplessness has been posited to elucidate the increased risk of internalizing symptoms in youths resulting from bullying victimization [33]. The significant mediating effects of both types of bullying victimization in the current study suggest that increased BMI is continuously correlated with the severity of bullying victimization, encompassing both traditional bullying and cyberbullying. This underscores the importance of prevention programs that target not only overweight/obese adolescents identified based on classification criteria but also those with an increased BMI as a vulnerable group susceptible to bullying victimization.

### 4.3. Sex as a Moderator

Previous research indicates that girls who are overweight or obese exhibit a greater preoccupation with weight and dietary patterns compared to their male counterparts facing similar weight challenges [34]. Consequently, it has been postulated that female adolescents might be more susceptible to the adverse impacts of overweight or obesity compared to their male counterparts [35]. Meanwhile, sex has also been observed to moderate the association between obesity and bullying victimization [36]. Therefore, it is reasonable to hypothesize that sex might exert a moderating effect on the relationship between increased BMI and the two types of bullying victimization (traditional bullying and cyberbullying). As expected, sex moderated the relationship between BMI and cyberbullying. According to the simple slope figure, being a boy appeared to function as a protective factor, whereas being a girl was identified as a risk factor in the relationship between BMI and cyberbullying victimization. Furthermore, most studies found that girls were more likely to be bullied online than boys [37,38,39,40,41,42]. One possible explanation for this finding is that cyberbullying, due to its characteristics such as anonymity and an unlimited audience, and the gender paradox hypothesis, which supposes that girls are more sensitive to the negative effects of cyberbullying victimization, can have a greater impact on girls [43].

Nevertheless, sex did not moderate the relationship between BMI and traditional bullying victimization. This may be related to the fact that the questionnaire in this study did not specifically categorize traditional bullying victimization. For example, in a Turkish study, the results showed that girls experienced a higher incidence of indirect bullying (e.g., gossiping) compared to boys, while boys reported a higher incidence of physical (e.g., damaging property) and verbal (e.g., teasing) bullying compared to girls [44]. Moreover, a study of Colorado’s statewide bullying prevention program showed that boys were more inclined to report instances of physical bullying compared to girls, while no gender disparities were identified in relation to verbal bullying [45].

### 4.4. Strengths and Limitations

The findings of this study have several strengths. First, to the best of our knowledge, it is the first to examine the moderation effects of traditional bullying victimization and cyberbullying victimization on the association between BMI and externalizing and internalizing scores, supporting the development of students’ physical and mental health. In addition, we used well-validated and widely used measures [46,47]. Despite these strengths, there are several shortcomings that should be considered in this study. Its cross-sectional design limited our ability to draw causal inferences from the associations among the variables. Thus, future research may adopt a longitudinal approach, which would be beneficial for elucidating the temporal relationships. Second, the data such as height and weight were based on adolescent self-reports and were subject to response bias. Thus, future studies should use multiple raters (e.g., parents and teachers) and apply additional sources of information or measures of BMI, bullying victimization, externalizing scores, and internalizing scores to further replicate the findings of this study [48]. Moreover, the study sample was restricted to participants from a single city in southern China, thereby limiting the generalizability of the findings to populations in other regions or countries. In addition, potential confounders such as smoking, alcohol consumption, and physical inactivity were not used in the final model, which may result in residual confounding. Lastly, the questionnaire in this study did not specifically categorize traditional bullying victimization, which may have led to non-significant results. Therefore, it is recommended that future research surveys categorize bullying victimization into specific types to better detect stronger associations.

### 4.5. Implications

We suggest that educators consider students’ experiences of victimization (both traditional bullying and cyberbullying) when dealing with students with increased BMI who have internalizing symptoms (emotional and peer problems) or externalizing symptoms (behavioral problems and hyperactivity), as both types of bullying victimization can play a role in these associations. Thus, it is necessary for school administrators to formulate relevant school rules to protect victims of bullying and provide psychological counseling. School teachers can intervene and verbally educate perpetrators of weight-based bullying. Meanwhile, academic institutions and public health researchers should actively promote education on fostering a positive body image, reducing weight stigma, and promoting an environment that supports the diversity of cultural dietary patterns [49]. This effort aims to cultivate a public discourse on obesity that aligns with current scientific findings [50].

## 5. Conclusions

Our study reveals that adolescents with an increased BMI, also known as obese and overweight adolescents, are significantly associated with externalizing and internalizing symptoms. Being a victim of either traditional bullying or cyberbullying mediates this relationship, and sex moderates the relationship between BMI and cyberbullying victimization. Thus, to reduce students’ internalizing and externalizing symptoms, it is advisable to contemplate prevention strategies or interventions that address both traditional bullying victimization and cyberbullying victimization when dealing with high-BMI students. Programs and interventions designed to prevent victimization (both traditional bullying and cyberbullying) and mitigate internalizing and externalizing symptoms should also take into account the influence of overweight and obesity. Additionally, girls should receive more attention regarding the issue of cyberbullying victimization.

## Figures and Tables

**Table 1:** Characteristics of study participants (*n* = 1486).

Characteristics	Total (*n* = 1486)	Mean or Percentage	Standard Deviations	Externalizing Symptoms (*n* = 292)	Internalizing Symptoms (*n* = 358)
SEX					
Girls	696	46.84		118	176
Boys	790	53.16		174	182
Grade					
7th	467	31.43		102	116
8th	508	34.19		85	104
10th	511	34.38		105	138
Age		14.10	1.30		
<14	555	37.35		105	125
14–16	913	61.44		184	228
17 or more	18	1.21		3	5
Traditional bullying victimization		14.15	6.59		
No	1202	80.89		66	124
Yes	284	19.11		226	234
Cyberbullying victimization		13.08	7.87		
No	1099	73.96		48	101
Yes	387	26.04		244	257
BMI		19.21	3.33		
Underweight	515	34.65		44	85
Normal weight	840	56.53		127	157
Overweight	100	6.73		91	85
Obese	31	2.09		30	31
Economy					
1	29	1.95		8	10
2	87	5.85		20	29
3	1193	80.28		228	271
4	142	9.56		25	34
5	35	2.36		11	14

Note: The total sample size is 1486. Missing data for 109 cases (7.3%) were imputed using maximum likelihood estimation.

**Table 2:** Descriptive statistics and correlations among study variables.

	1	2	3	4	5	6	7	8
Independent Variable								
1. BMI	1							
Dependent Variables								
2. Externalizing scores	0.59 **	1						
3. Internalizing scores	0.556 **	0.81 **	1					
Mediator Variables								
4. Traditional bullying victimization	0.61 **	0.48 **	0.45 **	1				
5. Cyberbullying victimization	0.73 **	0.60 **	0.55 **	0.49 **	1			
Moderator Variable								
6. Sex	0.04	0.05	−0.77 **	0.00	0.07 *	1		
Covariate Variables								
7. Age	0.05	0.00	0.05	−0.07	0.05	0.04	1	
8. Economy	0.03	0.00	−0.02	0.02	0.03	−0.03	−0.13 **	1

Note: * *p* < 0.05 and ** *p* < 0.01.

**Table 3:** Results of path analysis for two models.

Path	Model 1 (IS)						Model 2 (ES)					
	β	SE	*t*	*p*	LLCL	ULCL	β	SE	*t*	*p*	LLCL	ULCL
Direct path												
Age → TB	−0.096	0.021	−4.515	0.000	−0.139	−0.055	−0.096	0.021	−4.515	0.000	−0.139	−0.055
Age → CB	0.045	0.025	1.818	0.069	−0.004	0.094	0.045	0.025	1.818	0.069	−0.004	0.094
Age → IS	0.028	0.022	1.270	0.204	−0.015	0.072						
Age → ES							−0.022	0.021	−1.068	0.285	−0.063	0.018
Economy → TB	−0.007	0.021	−0.331	0.741	−0.049	0.034	−0.007	0.021	−0.331	0.741	−0.049	0.034
Economy → CB	0.002	0.023	0.106	0.915	−0.043	0.049	0.002	0.023	0.106	0.915	−0.043	0.049
Economy → IS	−0.030	0.025	−1.224	0.221	−0.080	0.017						
Economy → ES							−0.013	0.023	−0.586	0.558	−0.058	0.030
BMI → TB	0.612	0.024	25.878	0.000	0.563	0.656	0.612	0.024	25.878	0.000	0.563	0.656
BMI → CB	0.470	0.023	20.024	0.000	0.425	0.518	0.470	0.023	20.024	0.000	0.425	0.518
BMI → IS	0.399	0.028	14.006	0.000	0.340	0.453						
BMI → ES							0.409	0.031	13.235	0.000	0.345	0.467
TB → IS	0.175	0.025	6.905	0.000	0.127	0.226						
CB → IS	0.111	0.017	6.633	0.000	0.077	0.143						
TB → ES							0.157	0.030	5.151	0.000	0.099	0.219
CB → ES							0.194	0.026	7.451	0.000	0.144	0.246
BMI × SEX → TB	−0.046	0.035	−1.321	0.187	−0.114	0.024	−0.046	0.035	−1.321	0.187	−0.114	0.024
BMI × SEX → CB	−0.117	0.047	−2.478	0.013	−0.206	−0.021	−0.117	0.047	−2.478	0.013	−0.206	−0.021
SEX → TB	−0.019	0.021	−0.906	0.365	−0.061	0.023	−0.019	0.021	−0.906	0.365	−0.061	0.023
SEX → CB	−0.053	0.024	−2.243	0.025	−0.097	−0.005	−0.053	0.024	−2.243	0.025	−0.097	−0.005
Indirect path												
BMI → TB → IS	0.107	0.017	6.287	0.000	0.076	0.142						
BMI → CB → IS	0.052	0.008	6.816	0.000	0.037	0.067						
BMI → TB → ES							0.096	0.020	4.849	0.000	0.059	0.137
BMI → CB → ES							0.091	0.013	7.202	0.000	0.067	0.117

Note: TB: traditional bullying, CB: cyberbullying, IS: internalizing score, and ES: externalizing score.

**Table 4:** Analysis of the moderating effects of the two models.

Dependent Variable	Level	Model 1 and Model 2				
		Effect Size	SE	*p*	LLCL	ULCL
TB	Low (−SD)	1.139	0.095	0.000	0.945	1.321
	High (+1 SD)	1.329	0.105	0.000	1.108	1.521
	Difference	−0.190	0.143	0.185	−0.465	0.102
CB	Low (−1 SD)	4.254	0.674	0.000	3.015	5.648
	High (+1 SD)	7.119	0.962	0.000	5.253	9.070
	Difference	−2.865	1.190	0.016	−5.221	-0.489

**Table 5:** The moderated mediation effects of the models.

Path	Level	Effect Size	SE	*p*	LLCL	ULCL
BMI → TB → IS	Low (−1 SD)	0.127	0.022	0.000	0.088	0.173
	High (+1 SD)	0.149	0.024	0.000	0.105	0.198
	Difference	−0.021	0.016	0.194	−0.053	0.012
BMI → CB → IS	Low (−1 SD)	0.050	0.010	0.000	0.032	0.072
	High (+1 SD)	0.084	0.014	0.000	0.058	0.112
	Difference	−0.034	0.014	0.016	−0.061	−0.006
BMI → TB → ES	Low (−1 SD)	0.114	0.025	0.000	0.068	0.167
	High (+1 SD)	0.133	0.027	0.000	0.084	0.189
	Difference	−0.019	0.015	0.193	−0.047	0.011
BMI → CB → ES	Low (−1 SD)	0.088	0.017	0.000	0.058	0.124
	High (+1 SD)	0.147	0.024	0.000	0.103	0.197
	Difference	−0.059	0.025	0.018	−0.110	−0.010

## Data Availability

The dataset is available on request from the authors.

## Supplementary Materials

The following supporting information can be downloaded at: https://www.mdpi.com/article/10.3390/bs14060427/s1, File S1. Cross-Cultural School Children Study: Data instructions.
