# Supplementary material for: Association between Body Mass Index and Externalizing and Internalizing Symptoms among Chinese Adolescents: Mediating Role of Traditional Bullying and Cyberbullying Victimization
Source: Behav Sci (Basel). 2024 May 22;14(6):427. doi: 10.3390/bs14060427 (PMC11200949; doi:10.3390/bs14060427)
Supplement: Supplementary file 1 [file behavsci-14-00427-s001.zip › behavsci-2988362-supplementary.pdf]

Cross-Cultural School Children Study: Data instructions

The aim of this document is to help participating countries to entry study data into data set file in a consistent and similar way in each country to make comparison valid. Major part of the document is written about entering the data manually from paper questionnaires into electronic data file, but the same principles exists when the data is collected through optical reading process as well.

The main principle is that the data entry operator (DEO) enters to data set as much possible the same information what is found in paper questionnaire. Decisions about the "right" answers or the corrected values in unclear situations will be later made algorithmically to maintain maximum consistence between data sets from different countries.

Multiple answers

Sometimes a subject selects multiple options in a question where only single answer is expected. In these cases the right solution is to entry data as is, that is to include all concurrent choices. For example, if in SDQ the select answer is both 1 ("Not true") and 2 ("Somewhat true"), then the DEO is supposed to save both numbers separated with a semicolon: "1;2". If the field accepts only numeric input, then semicolon can be left out and the value is "12" (assuming "12" is not reserved for any other meaning in that context).

Answers between check boxes

Sometimes the mark is done between check boxes, but quite close to a specific box. In this case, the closer option is used. If the mark is pretty much in between two options, then clinically more severe or meaningful (more negative events, indicating more problems in child, school etc.) option is chosen. An example: Let's assume a subject has made crosses between boxes in SDQ1-3 like this:

	Not true		Somewhat true		Certainly true
(SDQ1) I try to be nice to other people. I care about their feelings.	☐	X	☐		☐
(SDQ2) I am restless, I cannot stay still for long.	☐		☐	X	☐
(SDQ3) I get a lot of headaches, stomach-aches or sickness	☐X		☐		☐

In this case, the appropriate value for SDQ1 item would be 1 ("Not true") and for SDQ2 item 3 ("Certainly true"), because they are more negative options, when the "direction" of the question is taken into account. SDQ3 would be 1 ("Not true") since it's clearly the nearest option in the form.

If answers are found both inside and outside check boxes then only answers inside are used.

Inappropriate forms

The whole form can be excluded in data entry process if it is clear that the form is filled mostly with inappropriate (fake/joke) answers. Number of such cases should be reported.

Missing values

Empty parts in form can be left empty in data as well; there is no need for a specific code for missing values (such as -999).

Open text

Open text fields, if any, can be entered into data in English. Long texts can be shortened, if it will not remarkably lose any information.

ID variables

Each subject should have a unique identification code in a data set to make possible later comparisons between information in data file and in paper questionnaire.

School information

We need school-level information as well. Dataset should include school name, school type (public vs. private school) and school area urbanity level (rural/urban) from your local perspective.

Note: If you participated the study the last time and used codes for schools (e.g. numbers, letters), please make sure that you use the same codes for the same schools this time, too.

Variable names

Variables can be named after their meaning and location in form. See appendix 1, which is a list of variables names and value labels in a sample data. Please follow the same naming schema as much as possible to make further data merging easier.

Note: Native Asian characters will not survive in data importation, or they may interfere the whole importing process, so please do not use them anywhere.

Variable value codes

Variables values should reflect the contents of questionnaire forms. See appendix 1, which is a list of variables names and value labels in a sample data.

Note: Native Asian characters will not survive in data importation, or they may interfere the whole importing process, so please do not use them anywhere.

Data files

The most preferable format for data set file are SAS data file (64-bit sas7bdat) and SPSS data file (sav). Plain text/ASCII (txt, csv, asc), Excel workbook (xls, xlsx), Access database (accdb), LibreOffice Calc spreadsheet (ods) are accepted as well.

Data transfer

Data transferring is done with an open-source enterprise file sync and share platform Seafile. Once your data is ready for transferring, upload it to the seafile using this link:
https://seafile.utu.fi/u/d/0321df03ea034eb19586/
Send an email to Joonas Laitinen (jralai@utu.fi), Emmi Heinonen (emmhei@utu.fi) and CC Sonja Gilbert (sonyle@utu.fi) afterwards to confirm.

Documents

We would like to receive following documents for each country:

1. Used questionnaire form in original language.
2. Used questionnaire form, English translation.
3. Code book for variable names and values.
4. Some additional general information about study:
   - When was the number of questionnaires sent and received?
   - What was the target group?
   - What was the source N and final N?

   That information could be written out like in this example: *The aim of the project was to study 8 grader school children in xxx part of the country X. All 1000 students of school grade 8 in city B in schools C and D were in the target group. In total 1000 paper questionnaires were sent to schools (500 to school C, 500 to school D) in 15th of Jan 2016 and 900 were received back by the end of March 2016 (school C 350, school D 450). From the 900 questionnaires 10 were excluded since they were empty (school C: n=4, school D: 6) and two were inappropriate (both in school C). Students of grade 8 in Jan 2016 are born in 2002.*

So please send us these documents and information, if you have not already done it.

Contact information

If you have any questions, comments or any other issues with the data, please do not hesitate to contact us.

Joonas Laitinen	Sonja Gilbert	André Sourander
jralai@utu.fi	sonja.gilbert@utu.fi	andre.sourander@utu.fi
Data manager	Researcher	Professor

Research Centre for Child Psychiatry
Lemminkäisenkatu 3/ Teutori 3.krs
20014 University of Turku, Finland

Based on a document by Lauri Sillanmäki
Updated by Emmi Heinonen (10.5.2023)

File S1. Variable names and labels. *Please notice that this an example only and most likely will not fit your data in every parts.*

Variable	*Label*
ID	Subject's identification code
Sex	Subject's gender ***1: Girl; 2: Boy; 3: Other***
Age	Subject's age in full years ***Num***
Grade	School grade ***Num***
School	School name ***Open***
School_type	School type ***1: Public, 2: Private***
Urbanity	School area urbanity category ***1: Rural; 2: Urban***
CIty	CIty ***Open***

MY FAMILY

1: (if selected)

Fam_struct1	I live with my biological parents
Fam_struct2	I live with my biological mother
Fam_struct3	I live with my biological father
Fam_struct4	I live with my biological parent and step parent
Fam_struct5	I live with my adoptive parents
Fam_struct6	I live with foster parents
Fam_struct7	I live with my grandparent or grandparents
Fam_struct8	I live with other relative (e.g. older sibling, uncle, aunt)
Fam_struct9	I live with two parents of the same sex
Fam_struct10	I live with other
Fam_struct10_who	I live with other, who? ***Open***
Fam_economic	How economically well off do you think your family is compared to other families?

1: Not well; 2: Not particularly well; 3: Fairly well; 4: Rather well; 5: Very well

MY BACKGROUND

0: No; 1:Yes

Backgr_A	I was born in Finland
Backgr_A_other	I was born where? ***Open***
Backgr_B	My native language is Finnish
Backgr_B_other	My native language is what? ***Open***
Backgr_C	My biological mother was born in Finland
Backgr_C_other	My biological mother was born where? ***Open***
Backgr_D	My biological father was born in Finland
Backgr_D_other	My biological father was born where? ***Open***

ACHES AND SLEEP

Aches_head — Have you experienced distracting headaches?

1: Hardly ever; 2: Less frequently; 3: At least once a month; 4:At least once a week

Aches_abdom — Have you experienced recurring abdominal pain?

1: Hardly ever; 2: Less frequently; 3: At least once a month; 4:At least once a week

Aches_sleep — Have you experienced problems with falling asleep or sleeping?

1: Never or less frequently than once a month; 2: Less than once a week; 3: Once or twice a week; 4: 3-5 times a week; 5: Every night or almost every night

Health_probl	Do you have an illness, disability or other health-related problem? ***0: No; 1: Yes***
Health_probl_what	If you answered yes, please specify? ***Open***

THOUGHTS ABOUT MY BODY

Body_height	My height is (cm)
Body_weight	My weight is (kg)

1: Not true; 2: Somewhat true; 3: Certainly true

Body_B1	I would like to be thinner
Body_B2	I exercise a lot to avoid gaining weight
Body_B3	I have been on a diet
Body_B4	I am afraid of getting fat
Body_B5	I have lost weight considerably over a short period of time due to not eating properly
Body_B6	I am not happy with my body
Body_B7	It terrifies me if I gain even a little weight
Body_B8	I am not always able to control my eating
Body_B9	I devour large amounts of food at one time
Body_B10	I have willfully vomited after having eaten
Body_B11	I have used pharmaceuticals to control my weight

ALCOHOL, CIGARETTES AND DRUGS

Intox_A	How often do you use alcohol? ***1: Never; 2: Once a month or more; 3: Once a week or more***
Intox_B	How often do you use alcohol to get drunk? ***1: Never; 2: Once a month or more; 3: Once a week or more***
Intox_C	How often do you smoke cigarettes or use other nicotine products? ***1: Never; 2: Rarely; 3: Weekly; 4: Daily***
Intox_D	Have you ever tried or used any illegal drugs? ***1: Never; 2: Once; 3: 2-4 times; 4: 5 times or more***

STRENGTHS AND DIFFICULTIES QUESTIONNAIRE

1: Not true; 2: Somewhat true; 3: Certainly true

sconsid	SDQ01: I try to be nice to other people. I care about their feelings
srestles	SDQ02: I am restless, I cannot stay still for long
ssomatic	SDQ03: I get a lot of headaches, stomach-aches or sickness
sshares	SDQ04: I usually share with others, for example CD's, games, food
stantrum	SDQ05: I get very angry and often lose my temper
sloner	SDQ06: I would rather be alone than with people of my age
sobeys	SDQ07: I usually do as I am told
sworries	SDQ08: I worry a lot
scaring	SDQ09: I am helpful if someone is hurt, upset or feeling ill
sfidgety	SDQ10: I am constantly fidgeting or squirming
sfriend	SDQ11: I have one good friend or more
sfights	SDQ12: I fight a lot. I can make other people do what I want
sunhappy	SDQ13: I am often unhappy, depressed or tearful

spopular	SDQ14: Other people my age generally like me
sdistrac	SDQ15: I am easily distracted, I find it difficult to concentrate
sclingy	SDQ16: I am nervous in new situations. I easily lose confidence
skind	SDQ17: I am kind to younger children
slies	SDQ18: I am often accused of lying or cheating
sbullied	SDQ19: Other children or young people pick on me or bully me
shelpout	SDQ20: I often offer to help others (parents, teachers, children)
sreflect	SDQ21: I think before I do things
ssteals	SDQ22: I take things that are not mine from home, school or elsewhere
soldbest	SDQ23: I get along better with adults than with people my own age
safraid	SDQ24: I have many fears, I am easily scared
sattends	SDQ25: I finish the work I'm doing. My attention is good
Exp_diff_A	SDQ26: Do you have difficulties in any of the following areas: emotions, concentration, behaviour or getting along with other people? ***1: No; 2: Yes – minor difficulties; 3: Yes – definite difficulties; 4: Yes – severe difficulties***
Exp_diff_B	SDQ27: How long have you experienced these difficulties? ***1: Less than a month; 2: 1–5 months; 3: 6–12 months; 4: Over a year***

1: Not at all; 2: Only a little; 3: Quite a lot; 4: A great deal

Exp_diff_C	SDQ28: Do the difficulties upset or distress you?
Exp_diff_D1	SDQ29: Do the difficulties interfere with your everyday life in home life?
Exp_diff_D2	SDQ30: Do the difficulties interfere with your everyday life in friendships?
Exp_diff_D3	SDQ31: Do the difficulties interfere with your everyday life in classroom learning?
Exp_diff_D4	SDQ32: Do the difficulties interfere with your everyday life in leisure activities?
Exp_diff_E	SDQ33: Do the difficulties make it harder for those around you (family, friends, teachers, etc.)?

SUICIDALITY

1: No, I have not; 2: Yes, once; 3: Yes, more than once

Sui_A	Have you intentionally hurt yourself for example by cutting or burning your skin?
Sui_B	Have you thought seriously about committing suicide?
Sui_C	Have you tried to commit suicide?

MY NEED FOR OUTSIDE HELP

Help_A	Within the past 6 months, have you at any point felt a need for outside help with your problems, feelings, behaviour or emotional trouble? ***1: No I have not felt the need; 2: I have considered getting outside help; 3: I have sought outside help***

1: (if selected)

Help_friend	If you have sought help, was it friends?
Help_relat	If you have sought help, was it relative?
Help_teach	If you have sought help, was it teacher?
Help_nurse	If you have sought help, was it school nurse?
Help_doct	If you have sought help, was it medical doctor?
Help_psych	If you have sought help, was it psychologist/school counsellor?
Help_relig	If you have sought help, was it religious or spiritual leader?
Help_coach	If you have sought help, was it coach in sports or other leisure activity?
Help_else	If you have sought help, was it someone else?
Help_else_who	If you have sought help from someone else, who was it? ***Open***

EXPERIENCES OF BEING BULLIED

1: Not at all; 2: Less than once a week; 3: More than once a week; 4: Most days

Exp_bul_A1	How often have you been bullied in school in the past six months?
Exp_bul_A2	How often have you been bullied away from school in the past six months?
Exp_bul_A3	How often have you been bullying others in school in the past six months?
Exp_bul_A4	How often have you been bullying others away from school in the past six months?
Exp_bul_B1	By whom you have been bullied - Girls
Exp_bul_B2	By whom you have been bullied - Boys
Exp_bul_B3	By whom you have been bullied - Adults
Exp_bul_B4	By whom you have been bullied - A group (e.g. a group of friends, a class etc.)
Exp_bul_C1	I have sibling(s) (half-siblings or similar) 0:No; 1: Yes
Exp_bul_C2	How often have you been bullied by a sibling at home in the past six months?
Exp_bul_C3	How often have you been bullying your sibling at home in the past six months?

CYBERBULLYIING

1: Never; 2: Less than once a week; 3: More than once a week; 4: Almost every day

Cyb_bul_A	During the past six months, how often have you been cyberbullied?
Cyb_bul_B	During the past six months, how often have you cyberbullied others?
Cyb_bul_C1	By whom have you been bullied - Girls
Cyb_bul_C2	By whom have you been bullied - Boys
Cyb_bul_C3	By whom have you been bullied - Adult women
Cyb_bul_C4	By whom have you been bullied - Adult men
Cyb_bul_C5	Person unknown to you
Cyb_bul_C6	A group (e.g. group of friends, school class etc.)

MY SCHOOL ENVIRONMENT

1: Never; 2: Sometimes; 3: Often; 4: Always

Env_A	I feel safe at school
Env_B	I can influence things at school
Env_C	I feel like I belong to the school community
Env_D	I enjoy going to school
Env_E	Violent situations or the risk of violence negatively affect my schoolgoing
Env_F	Teachers care about me
Env_G	Teachers treat me fairly
Env_H	I can speak to teachers about things I am concerned about
Env_I	I feel that homework burdens me

Env_J I get help with my studies at school if needed
Env_K Students of my class enjoy being together
Env_L My parents/guardians are interested in my schoolgoing
Env_M I get help with my problems at school from my parents/guardians if I need it
Env_N I think that the school facilities are cozy
Env_O Teachers or other adults at school try to stop bullying situations
Env_P Other students try to stop bullying situations

EXPERIENCES OF LONELINESS

Lonely_A How many close friends do you have? ***0:0 friends; 1: 1 friend; 2: 2 friends; 3: 3 or more friends***

Lonely_B During the past 12 months, how often have you felt lonely? ***1: Never; 2: Rarely; 3: Sometimes; 4: Most of the time; 5: Always***

EXPERIENCES OF THREATS

Threat_pand I am worried about pandemics, such as the COVID-19 pandemic. ***1: Not worried; 2: A little; 3: Moderately; 4: Very worried; 5: Extremely worried; 9: Prefer not to say***

0: No; 1: Yes; 9: Prefer not to say

Threat_pand_anx Do pandemics make you feel anxious?
Threat_pand_angry Do pandemics make you feel angry?
Threat_pand_afraid Do pandemics make you feel afraid?
Threat_pand_powerless Do pandemics make you feel powerless?

Threat_war I am worried about threat of war. ***1: Not worried; 2: A little; 3: Moderately; 4: Very worried; 5: Extremely worried; 9: Prefer not to say***

0: No; 1: Yes; 9: Prefer not to say

Threat_war_anx Does threat of war make you feel anxious?
Threat_war_angry Does threat of war make you feel angry?
Threat_war_afraid Does threat of war make you feel afraid?
Threat_war_powerless Does threat of war make you feel powerless?

Threat_climate I am worried that climate change threatens people and the planet. ***1: Not worried; 2: A little; 3: Moderately; 4: Very worried; 5: Extremely worried; 9: Prefer not to say***

0: No; 1: Yes; 9: Prefer not to say

Threat_climate_anx Does climate change make you feel anxious?
Threat_climate_angry Does climate change make you feel angry?
Threat_climate_afraid Does climate change make you feel afraid?
Threat_climate_powerless Does climate change make you feel powerless?

Threat_natural I am worried that natural disasters (wildfire, earthquakes, heavy rains, drought, tsunamis) threaten people and the planet.
1: Not worried; 2: A little; 3: Moderately; 4: Very worried; 5: Extremely worried; 9: Prefer not to say

0: No; 1: Yes; 9: Prefer not to say

Threat_climate_anx Do natural disasters make you feel anxious?
Threat_climate_angry Do natural disasters make you feel angry?
Threat_climate_afraid Do natural disasters make you feel afraid?
Threat_climate_powerless Do natural disasters make you feel powerless?

EXPERIENCES AND THREATS OF COVID-19 PANDEMIC

0: No; 1: Yes

Exp_covid_A Have you been quarantined by the health authorities due to COVID-19?
Exp_covid_B Have you been hospitalized because of COVID-19?
Exp_covid_C Has any of your family members or friends been hospitalized because of COVID-19?
Exp_covid_D Has any of your family members passed away because of COVID-19?

IMPACTS OF COVID-19 PANDEMIC ON YOUR FEELINGS, THOUGHTS AND BEHAVIOR

0: Not at all; 1: Rarely; 3: Sometimes; 5: Often

Feel_covid1 Do you think about it even when you don’t mean to?
Feel_covid2 Do you try to remove it from your memory?
Feel_covid3 Do you have waves of strong feelings about it?
Feel_covid4 Do you stay away from reminders of it (e.g. places or situations)?
Feel_covid5 Do you try not to talk about it?
Feel_covid6 Do pictures about it pop into your mind?
Feel_covid7 Do other things keep making you think about it?
Feel_covid8 Do you try not to think about it?

IMPACTS OF THE COVID-19 PANDEMIC ON YOUR DAILY LIFE

1: Not true; 2: Somewhat true; 3: Certainly true

Daily_covid1 My family has had financial difficulties
Daily_covid2 I have spent too much time on digital devices
Daily_covid3 The pandemic has had a negative effect on my school performance
Daily_covid4 My hobbies have been cancelled
Daily_covid5 There have been a lot of arguments in my family
